# Re-evaluation of all-plastic organic dye laser with DFB structure fabricated using photoresists

**DOI:** 10.1038/srep34741

**Published:** 2016-10-05

**Authors:** Naoto Tsutsumi, Saori Nagi, Kenji Kinashi, Wataru Sakai

**Affiliations:** 1 Faculty of Materials Science and Engineering, Kyoto Institute of Technology, Matsugasaki, Sakyo, Kyoto 606-8585, Japan; 2 Department of Macromolecular Science and Engineering, Graduate School of Science and Technology, Kyoto Institute of Technology, Matsugasaki, Sakyo, Kyoto 606-8585, Japan

## Abstract

Organic solid-state lasers (OSSLs) with distributed feedback structures can detect nanoscale materials and therefore offer an attractive sensing platform for biological and medical applications. Here we investigate the lasing characteristics, i.e., the threshold and slope efficiency, as a function of the grating depth in OSSL devices with distributed feedback (DFB) structure fabricated using photoresists. Two types of photoresists were used for the DFB structures: a negative photoresist, SU-8 2002, and a positive photoresist, ma-P 1275. The DFB structure was fabricated using a Lloyd-mirror configuration. The active layer was a rhodamine 6G-doped cellulose acetate waveguide. The threshold for the first order mode (*m*  = 1) was lower than that for the second and third order modes (*m* = 2, and 3). A low threshold of 27 μJ cm^−2^ pulse^−1^ (58 nJ) was obtained using SU-8 2002, with *m* = 1. The slope efficiency was evaluated as a function of grating depth for each mode and increased as the grating depth increased.

In the past two decades, organic solid-state lasers (OSSLs)^1^,^2^,^3^,^4^,^5^,^6^,^7^,^8^,^9^,^10^,^11^,^12^,^13^ have been developed extensively because of the easy fabrication of laser devices by spin-coating or solution casting, the low cost of device fabrication, and the wide variation of the lasing wavelength, ranging from the visible to near infrared regions, that can be selected depending on the laser dye used. Slope efficiency is an important measure to evaluate the performance of the laser devices. The slope efficiency is commonly determined by the slope between the pump (input) energy and output energy. A high brightness OSSL with a slope efficiency of 11% was achieved using a 4-(dicyanomethylene)-2-methyl-6-(4-dimethylaminostyryl)-4H-pyran (DCM)-based Vertical External-Cavity Surface-emitting Organic Laser (VECSOL)^14^. More recently, studies have shown that the organic thin film laser of the VECSOL can operate in single longitudinal mode (SLM) with the combination of a volume Bragg grating (VBG)^15^. Organic distributed feedback (DFB) dye lasers and distributed Bragg reflector (DBR) dye lasers also have specific features of SLM emissions. An OSSL possessing degradation recovery and based on polydimethylsiloxane as the host matrix was reported. Its durability increased 20.5-fold compared with that of an OSSL based on poly (methyl methacrylate) matrix^16^. OSSLs with DFB structure can detect nanoscale materials on DFB surface and therefore provide an attractive sensing platform for biological and medical applications^17^. A recent paper discusses the design and performance of organic DFB lasers in biosensor applications^17^.

Organic materials have features that enable the easy fabrication of corrugation structures for DFB and DBR resonators using photochromic materials, photoresists, and photopolymers. The height and width of the corrugation structures can be easily controlled using the illumination interference of the laser beams. The width of the corrugation structures, equivalent to the grating pitch, is related to the emission modes *m* = 1, 2, or 3. The height of the corrugation structures, equivalent to the grating depth, is related to the amount of feedback from the lasing light in a micro-cavity, which affects the lasing threshold and slope efficiency.

Although the significance of the grating depth and grating width on the lasing performance of DFB and DBR lasers has been well recognized, only a few studies have discussed these issues^18^,^19^,^20^.

In this report, we fabricated corrugated DFB structures using the same procedure described in our previous reports^21^,^22^, investigated the lasing characteristics of threshold and slope efficiency as a function of the grating depth, and compared our results with previous results^18^,^19^.

## Materials and Methods

### Materials

Two types of photoresists were used for the fabrication of a DFB: a negative type photoresist, SU-8 2002 (MicroChem), and a positive type photoresist, ma-P 1275 (Micro Resist Technology). Photoresist SU-8 2002 consisted of epoxy resin, photo acid generator, and cyclopentanone solvent. Photoresist ma-P 1275 consisted of novolac resin and the photoactive compound diazonaphthoquinone (DNQ). Rhodamine 6G (R6G) (Aldrich Chem.) was used as the laser dye. Cellulose acetate (CA, Aldrich Chem.) was used as the waveguide matrix in the laser device. Diacetone alcohol (DAA, Nacalai Tesque, Japan) was used as the solvent for CA. Polyvinyl alcohol (PVA, Nakalai Tesque, Japan) was used as an interlayer.

### Laser device with DFB structure

A solution of SU-8 2002/cyclopentanone (33/67 wt. %) was spin coated on a quartz substrate at 200–500 rpm for 10 s followed by 2000 rpm for 12 s. The obtained spin-coated film was pre-baked at 65 °C for 7 min and then at 95 °C for 14 min. An interference beam was illuminated on the SU-8 thin film using a Lloyd mirror configuration. To adjust the grating pitch, the thin film was set at an appropriate angle. A frequency-tripled Nd:YAG pulse laser delivering a 30 ps pulse at 355 nm with a 10 Hz repetition rate was used. After illumination, the SU-8 thin film was post-baked at 65 °C for 7 min and then at 95 °C for 7 min. The SU-8 thin film was developed in an SU-8 Developer for 1 min in an ultrasonic bath and then rinsed in 2-propanol for 30 s and dried before heating at 175 °C for 1 h.

A solution of ma-P 1275/ma-T 1030 (30/70 wt. %) was spin coated on a quartz substrate at 500 rpm for 10 s followed by 3000 rpm for 200 s. To remove the solvent, the ma-P 1275 thin film was heated on a hot plate at 100 °C for 10 min. An interference beam was illuminated on the ma-P 1275 thin film as described above. The ma-P 1275 thin film was developed in ma-D 331 for 1 min and rinsed with distilled water for 30 s. The obtained DFB structures were measured using an atomic force microscope (AFM, Nano-R, Pacific Technology).

PVA solution was spin coated on ma-P 1275 DFB to form a 0.2 μm PVA interlayer. The PVA interlayer protects the soluble ma-P 1275 DFB structure. A 0.5 wt. % R6G-doped cellulose acetate (CA) solution was spin coated on an SU-8 DFB structure or a PVA-coated ma-P 1275 DFB structure to fabricate an active laser waveguide device. The typical thickness of SU-8 and ma-P 1275 is 0.3 μm, and that of the active layer is 1.7 μm.

DFB structures was primarily fabricated using SU-8 photoresist except for the case that ma-P 1275 was used for the grating depth of 15 and 30 nm for *m* = 1. ma-P 1275 photoresist is preferred to fabricate deeper grating depth with wide incidence angle for *m* = 1.

### Laser emission

The frequency-doubled Nd:YAG pulse laser described above delivering a 30 ps pulse at 532 nm was used as the pumping laser source. For laser emission measurement, pumping laser beam was focused to a stripe shaped exciting beam with an area of 2.145 × 10^−3^ cm^2^ (a length of 33 × 10^−4^ cm and a width of 0.65 cm) using a cylindrical lens (f = 300 mm). The amplified spontaneous emissions (ASE) and lasing emissions from the edge of the waveguide were collected through a quartz optical fibre and monitored using a multi-channel analyser equipped with an Andor iDus charge coupled device (CCD) and gratings (1200 lines/500 nm). Pumping laser energy reflected by a half-mirror was monitored an Ophir photodiode type pyrometer PD10 with Nova II Display. Lasing beam energy emitted from the edge of the waveguide was monitored by an optical fibre coupled photodiode pyrometer (Ophir PD10-PJ with Nova II Display) equipped with an optical filter to cut an exciting beam in front^23^.

### Characterization

The absorption spectrum of a sample film was measured using a UV and visible spectrometer (UV-2100PC, Shimadzu, Japan). The photoemission spectrum of a sample film was recorded using a Shimadzu RF-1500 fluorophotometer. The film thickness was measured using an AFM.

The refractive indices of R6G doped CA, PVA, and ma-P 1275 films was measured at 632.8 nm and 830 nm using an m-line technique of the prism coupling method. A prism and waveguide were coupled with an air-gap. A He-Ne laser at 632.8 nm and a laser diode at 830 nm were used as laser sources for the prism coupling method. Refractive indices of R6G doped CA, SU-8 2002, PVA, and ma-P 1275 films were measured in TE mode at 632.8 and 830 nm are shown in Table 1. Index of refraction at some wavelength *n*(λ) was evaluated using a one-oscillator Sellmeier-dispersion formula as follows:


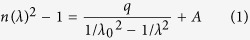


where *q* is a measure of the oscillator strength, *A* is a constant containing the sum of all other oscillators, and *λ*_0_ is a absorption wavelength of dominant oscillator. The value of *λ*_0_ for each film is shown in Table 1. The values of *q* and *A* were calculated for each film with the values of refractive index and *λ*_0_ listed in Table 1. Refractive index of lasing wavelength region around 590 nm, *n* = 1.48 for R6G doped CA, *n* = 1.59 for SU-8 2002, *n* = 1.62 for ma-P 1275, and *n* = 1.54 for PVA were evaluated.

## Results and Discussion

### Waveguide modes

**Figure 1:**
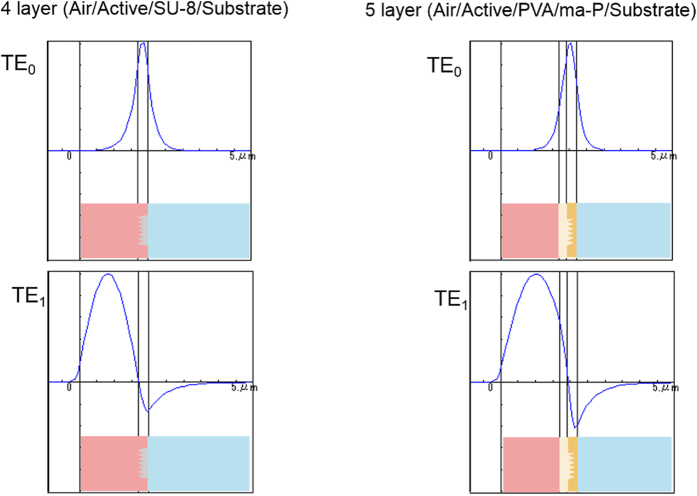
Optical confinement (blue curve) in four-layer and five-layer waveguides. Optical confinement was calculated using an NL-guide waveguide simulation software. For both waveguides, light is confined in photoresists with higher refractive indices for the TE_0_ mode, whereas light is confined in the active layer and gain medium for the TE_1_ mode.

The simplest waveguide laser is a three-layer structure that consists of: air, a core with a gain medium and a clad layer with relief grating structures. In the presented waveguide laser device with DFB structures, four- and five-layer waveguides were employed. The four-layer waveguide consisted of air (*n* = 1)/R6G doped CA gain medium (*n* = 1.48)/SU-8 2002 (*n* = 1.59) with a grating structure/Quartz substrate (*n* = 1.46). The five-layer waveguide consisted of air (*n* = 1)/R6G doped CA gain medium (*n* = 1.48)/PVA intermediate layer (*n* = 1.54)/ma-p 1275 (*n* = 1.62) with a gain medium/Quartz substrate (*n* = 1.46). The refractive index of the waveguide core, R6G doped CA, is 1.48, which is lower than those of the SU-8 2002 (*n* = 1.59), ma-P 1275 (*n* = 1.62), and PVA interlayer (*n* = 1.54). The confinement of light is calculated using a waveguide simulated by an NL-guide software. The confinement of light in the waveguide is shown in Fig. 1. In the TE_0_ waveguide mode, light is confined in the SU-8 2002 layer because of the high refractive index of SU-8 2002, 1.59. Whereas, in the TE_1_ waveguide mode, light is confined in the CA active layer with the R6G laser dye possessing a refractive index of 1.48.

The same type of situation is evaluated in a five-layer waveguide. In a similar manner, light is confined in PVA and ma-P 1275 in the TE_0_ waveguide mode, whereas it is confined in the active layer in the TE_1_ mode. The thicknesses of the PVA and ma-P 1275 layers are 0.2 and 0.3 μm, respectively.

Thus, the preferential waveguide mode for the present DFB laser is determined to be the TE_1_ mode for both photoresist waveguides.

### Fabrication of grating structures

**Figure 2:**
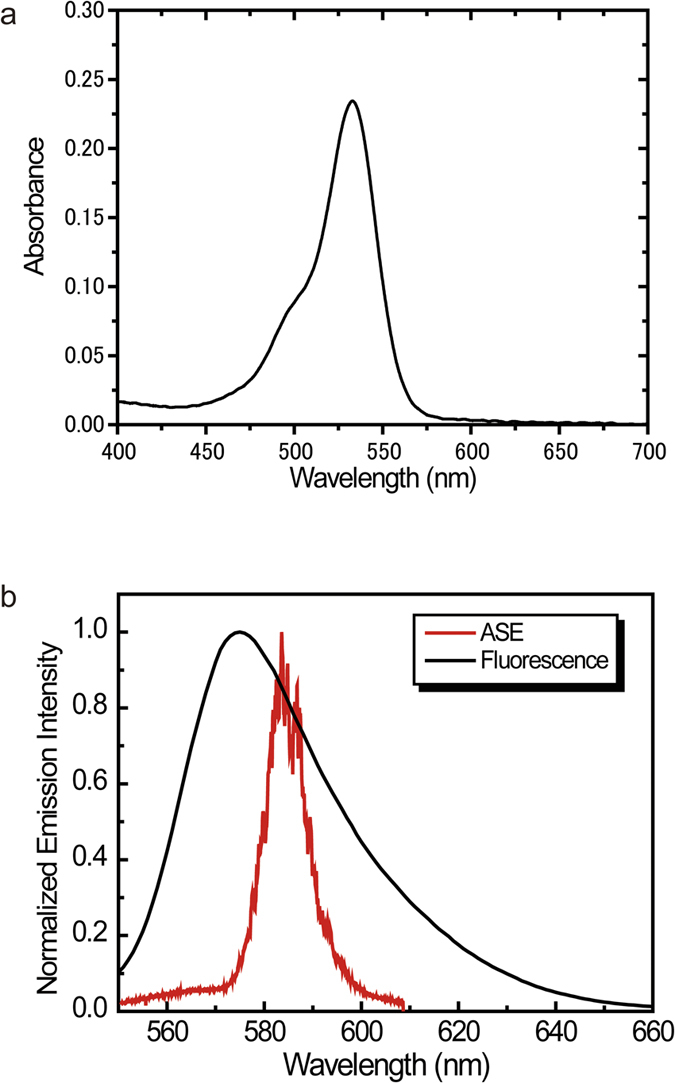
Absorption, photoluminescence, and ASE spectra of Rhodamine 6G in a CA thin film. (**a**) Absorption spectrum. (**b**) Fluorescence and ASE spectra. Fluorescence broadens from 550 to over 650 nm. ASE is in the range from 570 to 610 nm.

**Figure 3:**
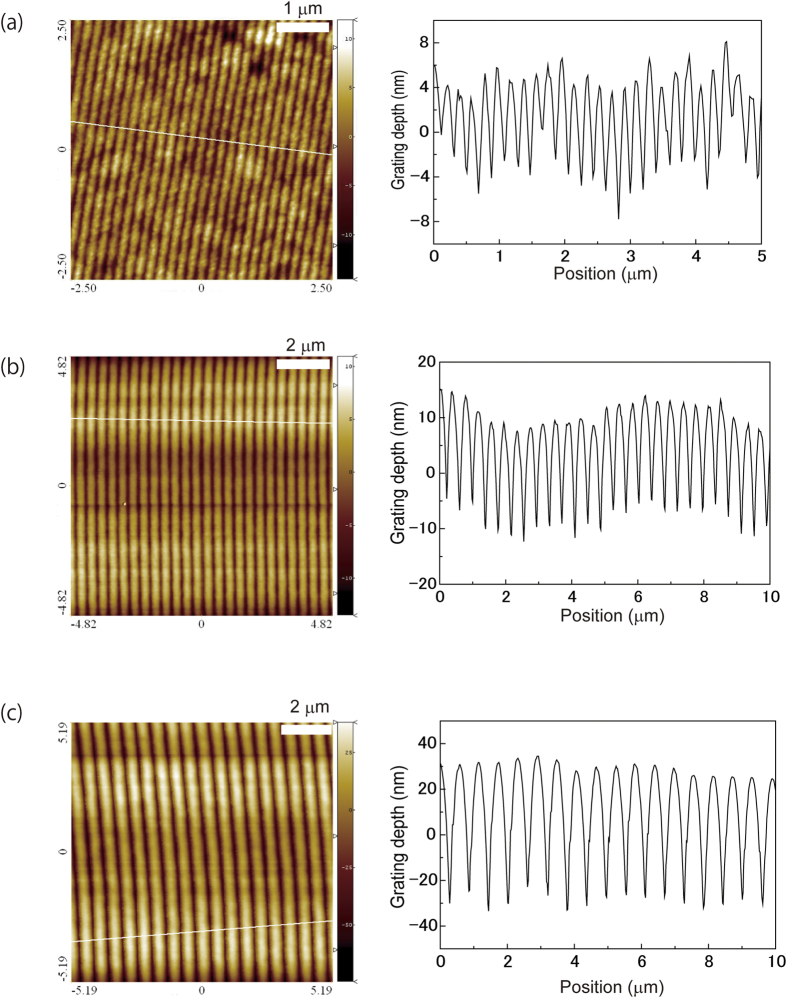
AFM images and grating depths of corrugation structures fabricated in SU-8 photoresist by interference beams. Grating depth is measured along a white line in each AFM image. (**a**) A grating pitch of 199 nm which corresponds to lasing mode *m* = 1, (**b**) a grating pitch of 390 nm which corresponds to lasing mode *m* = 2, and (**c**) a grating pitch of 590 nm which corresponds to lasing mode *m* = 3.

In DFB lasers, the properties of the grating structures (grating depth and grating pitch) are important for lasing performance. The grating pitch is directly related to the mode number for diffraction. Figure 2 shows the absorption, fluorescence, and amplified spontaneous emission (ASE) spectra of 0.5 wt% R6G in the CA film. The thickness of the film was 1.49 μm, which was determined using a Lambert-Beer law. Extinction coefficient of R6G is 116,000 cm^−1^ M^−1^ at peak wavelength^24^. Density of CA is 1.3 kg dm^−3^. Molecular weight of R6G is 479.02 g mol^−1^. Absorbance of spin-coated film was 0.234 at peak wavelength. The ASE ranges from 570 to 610 nm. Lasing occurs in the ASE range. Thus the grating pitch equivalent to the resonance length of the micro-cavity in the DFB structures should be determined. The grating pitch, which is the same as the resonance length of the micro-cavity *L* in DFB structures is calculated as follows:


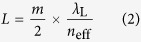


where *m* is the diffraction mode, *λ*_L_ is the lasing wavelength, and *n*_eff_ is the effective refractive index. Typically, the grating pitch is 200 nm for *m* = 1, 400 nm for *m* = 2, and 600 nm for *m* = 3, with *λ*_L_ = 600 nm and *n*_eff_ = 1.5. There are several ways to fabricate the grating in photoresists: electron lithography, direct laser writing, and by the interference of laser beams. In this report, the interference of two beams method was employed. To fabricate grating pitches of 200, 400, and 600 nm, the incidence angles of the interference beam were 62.6°, 26.3°, and 17.2°, respectively. Figure 3 shows AFM images of fabricated gratings in SU-8 photoresist for the mode numbers (a) *m* = 1, (b) *m* = 2, and (c) *m* = 3. The grating depth profile for each mode is also shown in Fig. 3. The grating depth becomes shallower with wider incidence angles.

### Lasing threshold and slope efficiency

**Figure 4:**
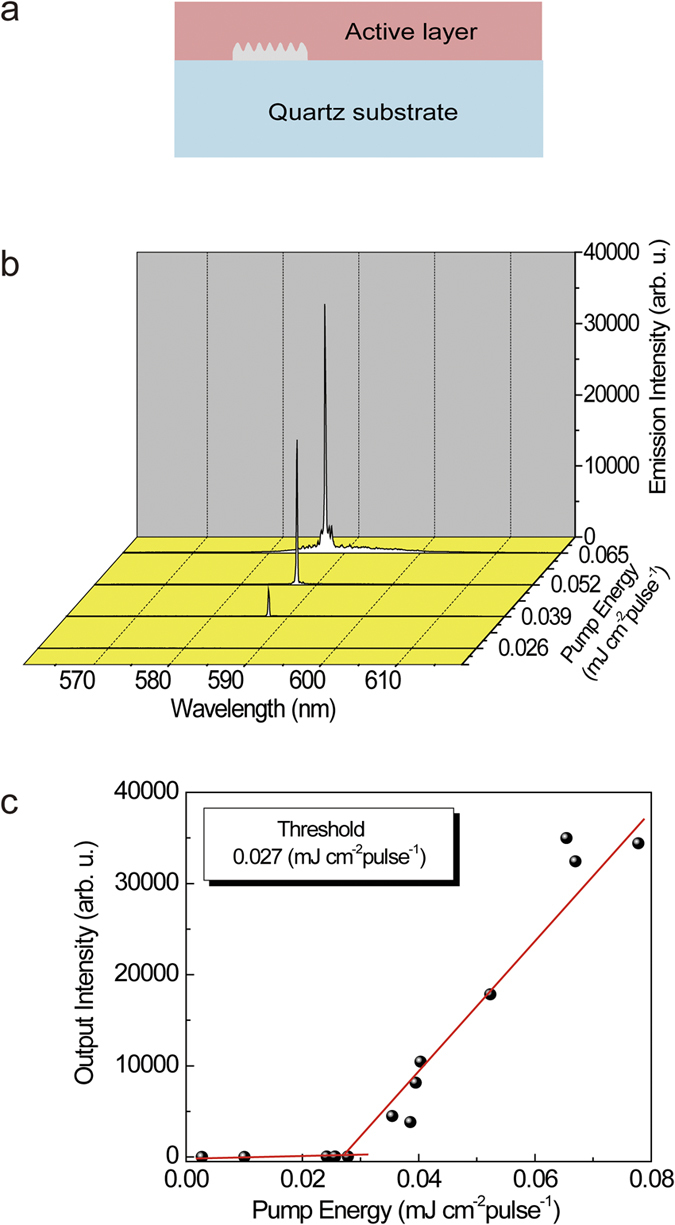
(**a**) Schematic diagram of DFB laser device. Corrugation structures of SU-8 photoresist were fabricated 6.5 mm inside the edge. (**b**) Lasing peak profile of DFB laser with mode *m* = 1. Emission wavelength is 587.4 nm. Lasing occurred at 587.4 nm with the DFB structures fabricated using the incidence angle of 65.5°. (**c**) Plots of emission intensity as a function of pump energy. Intersect of the two lines gives the lasing threshold.

A typical DFB laser device is shown in Fig. 4(a). The corrugated structure was fabricated in SU-8 photoresist and it was located 6.5 mm inside the substrate edge using the Lloyd mirror technique.

**Figure 5:**
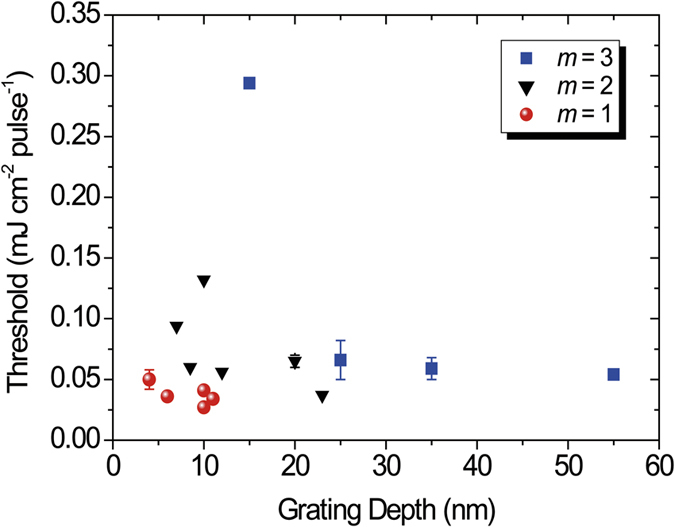
Plots of lasing threshold as a function of grating depth for *m* = 1 (red circle plots), *m* = 2 (black inverse triangle plots), and *m* = 3 (blue square plot). A lower mode number gives a lower threshold. All corrugated structures are fabricated by SU-8 photoresist.

The threshold and slope efficiency of DFB lasing are measured to evaluate the lasing performance of the DFB lasers. The lasing threshold was determined by the pump energy at which a narrow lasing peak appeared. Figure 4(b) is a typical lasing peak profile for DFB lasing when the pump energy is changed from 26 to 65 μJ cm^−2^ pulse^−1^ for the mode *m* = 1. The lasing wavelength is 587.4 nm and the lasing width is <0.2 nm. The emission intensity is plotted as a function of the pump energy in Fig. 4(c). The lasing intensity increases linearly with increasing pump energy. The lasing threshold is determined by the intersection of two lines, as shown in Fig. 4(c). A lasing threshold of 27 μJ cm^−2^ pulse^−1^ (58 nJ) is evaluated from the intersection of two lines. Figure 5 shows the dependence of the lasing threshold on the grating depth for the modes *m* = 1, *m* = 2, and *m* = 3. All corrugated structures were fabricated by SU-8 photoresist. The lasing threshold for *m* = 1 is lower than that for *m* = 2 and *m* = 3. This result is reasonable because significant emission loss from the waveguide due to the radiation loss (radiation mode) should be considered for higher modes *m* ≥ 2.

**Figure 6:**
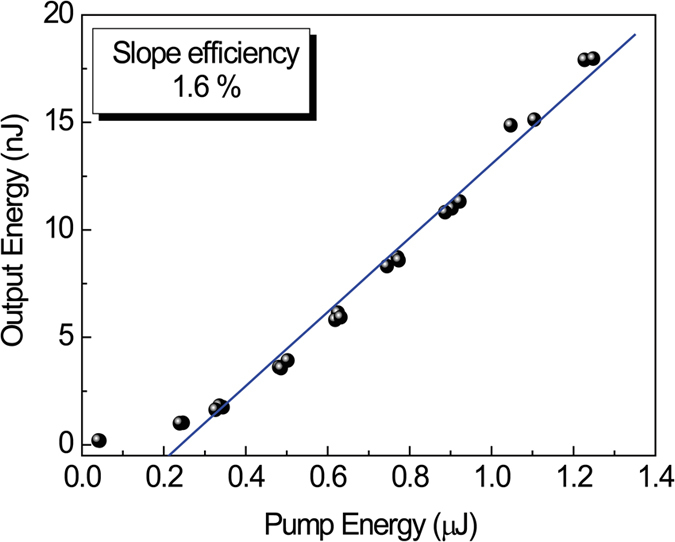
Plots of pump energy and emitted (output) energy. Blue line is a slope efficiency of 1.6%. Corrugated structure is fabricated in SU-8 photoresist.

The slope efficiency is determined by the slope between the pump (input) energy and output energy. The output energy is plotted as a function of the input energy for the mode *m* = 1 with a grating height of 15 nm and a grating pitch of 189 nm, as shown in Fig. 6. The slopes of the plots give the slope efficiency, which is 1.6% in this case.

**Figure 7:**
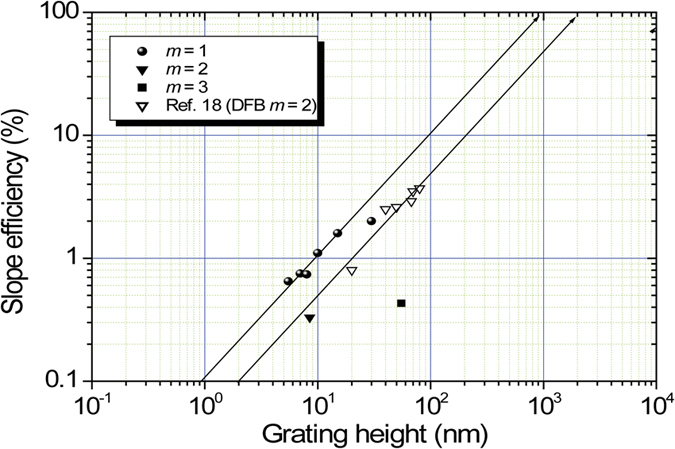
The plots of slope efficiency as a function of grating depth. The black circle is the slope efficiency for lasing mode *m* = 1, the black inverse triangle is for *m* = 2, and the black square is for *m* = 3. The white inverse triangle is taken from ref. 18 and replotted. Gain matrix is F8BT doped by MEH-PPV.

For feedback lasing using DFB structures, the grating depth (height) is important to determine the feedback of light in the feedback cavity. Thus the slope efficiency is investigated as a function of the grating depth (height). The grating depth was varied with the change in the period of illumination. We can vary the grating depth (height) between 5 and 30 nm for the mode *m* = 1 using an interference technique with a grating pitch between 189 and 200 nm. Su-8 photoresist was used except for the case that ma-P 1275 photoresist was used for the grating depth of 15 and 30 μm for *m* = 1. A slope efficiency of 0.33% for the mode *m* = 2 was measured for the sample with a grating pitch of 391 nm and a grating depth of 8.5 nm. A slope efficiency of 0.43% for the mode *m* = 3 was measured for the sample with a grating pitch of 590 nm and a grating depth of 55 nm. The slope efficiencies are plotted together as a function of the grating depth in Fig. 7. Several recent papers have also discussed the relationship between the slope efficiency and the grating depth^18^,^19^ in DFB lasing. Recent results published by other researchers^18^ are plotted in Fig. 7 as a reference. The laser active material was the polyfluorene derivative poly[(9,9-dioctylfluorenyl-2,7-diyl)-co-(1,4-benzo{2,10,3}-thiadiazol)] (F8BT) doped with 15 wt. % of poly[2-methoxy-5–(20-ethyl-hexyloxy)–1,4-phenylene vinylene] (MEH-PPV). The grating pitch was 410 nm^18^, so the lasing mode was assumed to be *m* = 2. A lasing slope efficiency of less than 1% was reported in an R6G-doped SU-8 photoresist in which the grating structure with a grating depth of 80 nm and a grating pitch of 550 nm (equivalent to the mode *m* = 3) was fabricated by two-photon laser fabrication^19^. These results are in good agreement with the present results for the mode *m* = 3.

The coupling of waves is defined by the coupling coefficient *κ* as follows:


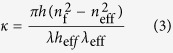



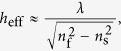


where *h* is equivalent to the grating height, *h*_eff_ is the minimum effective film thickness of the fundamental mode, *n*_f_ is the refractive index of the active layer and *n*_s_ is the refractive index of the substrate^18^.

The feedback gain *G* is the product of the coupling coefficient and the resonance length of the micro-cavity *L*^25^,^26^,^27^. *L* increases linearly with the diffraction mode for lasing *m*.


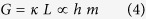


Thus, larger values of *h* and deeper grating depths result in larger feedback gains, which lead to efficient lasing performance and a higher slope efficiency. The straight line in Fig. 7 gives the linear relation between the slope efficiency and grating depth. If the feedback gain is parallel to the slope efficiency, a grating depth of 100 nm achieves a slope efficiency of 10% and a 1000 nm grating depth achieves a 100% slope efficiency for the mode *m* = 1. Direct laser writing is promising for the fabrication of high aspect ratio grating structures to achieve a high lasing slope efficiency.

We must also consider the contradictory relationships: a higher diffraction mode leads to a larger optical loss due to the radiation loss of the feedback light from the waveguide (radiation mode). In other words, for the diffraction mode higher than *m* = 2, part of the feedback light is radiated from the waveguide as a radiation mode, which diminishes the lasing performance. This situation can be predicted from Fig. 7.

## Conclusions and Outlook

We investigated the lasing characteristics, i.e., threshold and slope efficiency, as a function of the grating depth in OSSL devices with a DFB resonator fabricated using photoresists. The threshold for the first order mode (*m* = 1) was lower than that for the second and third order modes (*m* = 2, and 3). A low threshold of 27 μJ cm^−2^ pulse^−1^ (58 nJ) was obtained using SU-8 2002 with the mode *m* = 1. The slope efficiency was evaluated as a function of the grating depth for each mode. The slope efficiency increased with increasing grating depth, which can be explained by the increase in the coupling gain. In the higher modes greater than 2, the radiation loss reduced the lasing performances. Thus the mode number *m* = 1 gives the highest lasing performance (lowest threshold and highest slope efficiency). The present report assumes that a grating depth of 100 nm achieves a 10% slope efficiency and a grating depth of 1000 nm achieves a 100% slope efficiency for the mode *m* = 1.

## Additional Information

**How to cite this article**: Tsutsumi, N. *et al.* Re-evaluation of all-plastic organic dye laser with DFB structure fabricated using photoresists. *Sci. Rep.* **6**, 34741; doi: 10.1038/srep34741 (2016).

## Figures and Tables

**Table 1:** Refractive indices (RI) and *λ*_0_ for R6G doped CA, SU-8 2002, ma-P 1275, and PVA films.

Film	RI @ 632.8 nm	RI @ 830 nm	*λ*_0_ (nm)
R6G doped CA	1.4736	1.4712	280
SU-8 2002	1.587	1.578	291
ma-P 1275	1.6138	1.6107	344
PVA	1.5391	1.5264	200
